# AlncRNA HULC as an effective biomarker for surveillance of the outcome of cancer: A meta-analysis

**DOI:** 10.1371/journal.pone.0171210

**Published:** 2017-02-01

**Authors:** Xiaoliang Chen, Jinbo Lin, Yi Liu, Ji Peng, Yong Cao, Zhan Su, Tieqiang Wang, Jinquan Cheng, Dongsheng Hu

**Affiliations:** 1 Shenzhen Guangming district center for disease control and prevention, Shenzhen, Guangdong, P.R. China; 2 Longgang district central hospital of Shenzhen, Shenzhen, Guangdong, P.R. China; 3 Shenzhen Center for Chronic Disease Prevention, Shenzhen, Guangdong, P.R. China; 4 Department of Molecular Biology, Shenzhen Center for Disease Control and Prevention, Shenzhen, Guangdong, P.R. China; 5 School of Medicine, Shenzhen University, Shenzhen, Guangdong, P.R. China; University of South Alabama Mitchell Cancer Institute, UNITED STATES

## Abstract

**Purpose:**

High expression of highly upregulated in iver cancer (HULC) has been found associated with increased metastasis and poor prognosis with cancer. This meta-analysis aimed to determine the pooled effect of HULC on metastasis and prognosis of cancers.

**Method:**

The studies were accessed using multiple databases. RevMan5.3 and STATA14.0 were used to estimate pooled effects, the heterogeneity among studies, and publication bias for the association of HULC and overall survival (OS).

**Results:**

A total of 9 studies of 966 cancer patients were included. Risk of lymph node metastasis was increased with high versus low HULC expression (pooled odds ratio [OR] = 4.83, 95% confidence interval [CI] 1.59–14.63) as was distant metastasis (pooled OR = 5.44, 95% CI 2.33–12.74). Furthermore, OS time was shortened with high HULC expression (pooled hazard ratio [HR] = 1.48, 95% CI 1.03–2.12), especially in Chinese patients (pooled HR = 2.04, 95% CI 1.55–2.68), and risk of recurrence was increased (pooled OR = 6.68, 95% CI 2.77–16.13).

**Conclusion:**

HULC might be a potential biomarker for therapy and prognosis surveillance in cancers.

## Background

With the development of transcriptome and genome sequencing technologies, many long noncoding RNAs (lncRNAs) have been detected and reported,[1, 2] and found involved in almost all aspects of gene expression including protein translation and stability.[3] These lncRNAs have shown aberrant expression in tumor tissues and patient serum. Abundant evidence has shown lncRNAs involved in many cellular cancer pathways, including the regulation of gene expression, protein localization, and formation of essential protein complex substructures [4–6]. Furthermore, lncRNAs such as MALAT1, HOTAIR, and H19 were found to act as tumor suppressor genes or oncogenes in cancer development [7–9].

Recently, a novel lncRNA, highly upregulated in liver cancer (HULC), has attracted widespread attention. HULC has been found dysregulated in various human tumors, such as hepatocellular carcinoma, colorectal carcinoma, osteosarcoma, gastric cancer, and diffuse large B-cell lymphoma [10–13]. Abnormal expression of HULC in cancerous tissue or serum has confirmed it as an important player in tumorigenesis of cancers. A high level of HULC was found associated with metastasis and prognosis of cancers [10–13]. HULC might be a diagnostic biomarker and prognostic factor of cancers. However, the effect of HULC on cancer prognosis is controversial, and no meta-analysis has investigated the relationship between HULC expression and prognosis.

The present meta-analysis aimed to explore the association of HULC level and clinical outcome of cancer patients to further determine the biomarker role of HULC in metastasis and prognosis of cancers.

## Material and methods

### Literature search strategy

Reports of studies in English or Chinese on the role of HULC in the development of human cancer were searched in PubMed, EMBASE, the Cochrane Library, China National Knowledge Infrastructure, and Wanfang databases with the key words (“hepatocellular carcinoma up-regulated long non-coding RNA” or “HULC” or “HCCAT1” or “LINC00078” or “NCRNA00078”). The last search date was September 7, 2016. References of retrieved papers and conference reports were also searched to identify relevant studies. The preferred reporting items for systematic reviews and meta-analyses (PRISMA) was listed in S1 Table.

### Selection criteria

After duplicates were removed, titles and abstracts of articles were checked by 4 authors (JL, YL, JP, and YC). The full text of eligible articles was retrieved. Eligible articles had the following criteria: 1) the expression of HULC was analyzed by metastasis or survival time, 2) patients were divided by high and low expression of HULC, 3) the effects for metastasis or survival were provided or could be calculated from the available data; for the metastasis, odd ratios (ORs) of lymph node metastasis [LNM] and distant metastasis [DM] were considered; for survival, hazard ratios (HRs) of overall survival [OS], disease-free survival [DFS], event-free survival [EFS] and progression-free survival [PFS] were considered; and 4) the expression of HULC was tested in cancer tissue or serum by RT-PCR or fluorescence *in-situ* hybridization. Studies not fulfilling the criteria, reviews, animal/cell-line studies, and case reports were excluded. Furthermore, if more than 1 study of the same cohort was published, only the most recent publication was included.

### Data extraction and quality assessment

Three authors (XC, ZS, TW) extracted the following data by using an extraction form: first author’s name, published year, region of cohort, sample size, cancer type, method to test HULC, cases in each expression group (high/low), cases of recurrence or metastasis type (LNM, DM) in each group, and survival results (OS, DFS, EFS and PFS). Furthermore, the reference for all effects (ORs or HRs) was reformatted as low HULC expression, and the multivariate analysis effects were used for pooled analysis. The quality of each eligible study was assessed by the Newcastle-Ottawa Scale (NOS), consisting of selection, outcome and comparability, with scores from 0 to 9. A study with NOS score ≥6 was considered at high quality. Consensus in extraction was resolved by discussion and with 2 other investigators (JC, DH) if needed.

### Statistical methods

This meta-analysis involved use of Review Manager 5.3 (Cochrane network). Inconsistency (I^2^) and Q tests (chi-square test) were used to test for heterogeneity among eligible studies. If statistical heterogeneity was found (*P*_*Q*_*<*0.05, *I*^*2*^*>*50%), a random-effects model based on the work of Der Simonian and laird was used to estimate the pooled OR and HR. Otherwise, a fixed-effects model according to the Mantel-Haensed method was used [14]. HRs and 95% CIs for OS were extracted from Kaplan-Meier curve analysis by using Engauge Digitizer 4.1[15] because HRs and 95% CIs were not provided directly in some studies. Moreover, Begg’s and Egger’s tests were used to assess publication bias, and trim and fill analysis was used to adjust the pooled effects obtained by STATA 14.0[16]. All tests were two-sided and *P<*0.05 was considered statistically significant.

## Results

### Characteristics of eligible studies

**Fig 1:**
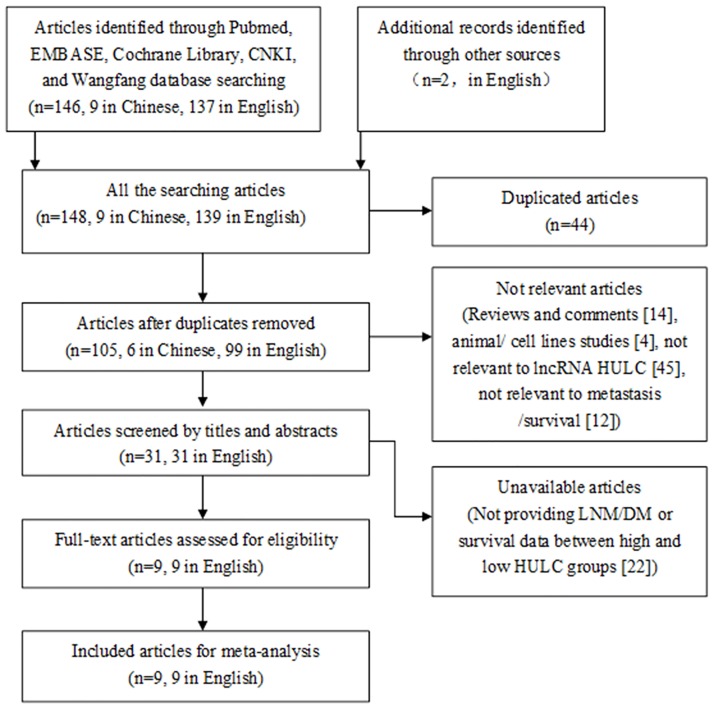
Flow of the studies in the meta-analysis. HULC, highly upregulated in liver cancer.

The literature search resulted in 9 studies eligible for the meta-analysis [11, 17–21, 13, 22, 23] (Fig 1), 7 from China and 2 from Brazil and Korea. The studies involved a total of 966 cancer patients, with mean sample size of 107.3 patients (range 33 to 240). Six different types of cancer were evaluated: hepatocellular carcinoma, osteosarcoma and gastric cancer (n = 2 each), diffuse large B-cell lymphoma, pancreatic cancer, and colorectal carcinoma (n = 1 each). The level of HULC was detected in tumor tissue or serum by RT-PCR and the negative control was adjacent noncancerous tissues, healthy tissues, and healthy serum. Furthermore, the group cut-off value for clinic outcome determined by the original research depended on the median/mean value of HULC level or ROC analysis. The main characteristics of each study are summarized in Table 1 and NOS scores indicate good methodological quality of the component studies with range of 7 to 8 (Table 2).

**Table 1:** Basic data for all included studies in the meta-analysis.

First author	Year	Country	Cohort sample size	Cancer type (derived)	Detection method	HULC expression							Survival		quality score
						Low/ high	Low			High			Variance Analysis	Outcomes	
							LNM	DM	Recurrence	LNM	DM	Recurrence			
Uzan VR, et al	2016	Brazil	33	Osteosarcoma (tissue)	RT-PCR	21/12	NR	NR	NR	NR	NR	NR	Univariate	OS, EFS	7
Yang Z, et al.	2015	Korea	240	Hepatocellular Carcinoma (tissue)	RT-PCR	NR	NR	NR	NR	NR	NR	NR	Multivariateanalysis	OS, DFS	7
Sun XH, et al.	2015	China	78	Osteosarcoma (tissue)	RT-PCR	39/39	NR	5/34	NR	NR	16/23	NR	Multivariateanalysis	OS	7
Li SP, et al.	2016	China	38	Hepatocellular Carcinoma (tissue)	RT-PCR	15/23	NR	1/14	5/10	NR	8/15	16/7	Univariate	OS	7
Jin C, et al.	2016	China	54	gastric cancer (serum)	RT-PCR	27/27	25/27	2/50	NR	34/14	9/39	NR	Univariate	OS	7
Peng W, et al.	2014	China	304	pancreatic cancer (tissue)	RT-PCR	92/212	23/69	NR	79/13	157/55	NR	208/4	Multivariateanalysis	OS	8
Peng W, et al.	2016	China	142	Diffuse large B-cell lymphoma (lymph node)	RT-PCR	47/95	NR	NR	NR	NR	NR	NR	Multivariateanalysis	OS, PFS	8
Zhang Y, et al.	2016	China	42	gastric cancer (serum)	RT-PCR	20/22	NR	NR	NR	NR	NR	NR	Univariate	OS	8
Yang XJ, et al.	2016	China	35	colorectal carcinoma (tissue)	RT-PCR	12/23	NR	NR	NR	NR	NR	NR	Univariate	OS	8

Abbreviations: LNM, lymph node metastasis; DM, distant metastasis; OS, overall survival; DFS, disease-free survival; EFS, event-free survival; PFS, progression-free survival; NR, no report

**Table 2:** Details of the Newcastle-Ottawa Scale score for included studies.

NOS items	Uzan VR, et al	Yang Z, et al.	Sun XH, et al.	Li SP, et al.	Jin C, et al.	Peng W, et al., 2014	Peng W, et al., 2016	Zhang et al.	Yang et al.
Representativeness of the exposed cohort	1	1	1	1	1	1	1	1	1
Selection of the non-exposed cohort	1	1	1	1	1	1	1	1	1
Ascertainment of exposure	1	1	1	1	1	1	1	1	1
Demonstration that outcome of interest was not present at the start of the study	1	1	1	1	1	1	1	1	1
Comparability of cohorts on the basis of the design or analysis	1	1	1	1	1	1	1	1	1
Assessment of outcome	1	1	1	1	1	1	1	1	1
Follow-up was long enough for outcomes to occur	1	1	1	1	0	1	1	1	1
Adequacy of follow-up of cohorts	0	0	0	0	0	1	1	1	1
Total score	7	7	7	7	7	8	8	8	8

### Association between HULC and metastasis

#### Association between HULC and LNM

**Fig 2:**
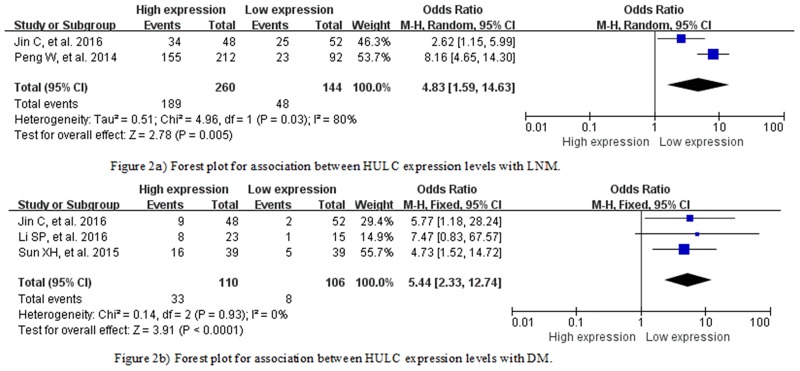
**a)** Forest plot of association between HULC expression and lymph node metastasis (LNM). **b)** Forest plot of association between HULC expression and distant metastasis (DM). **Abbreviations:** CI, confidence interval; M-H, Mantel-Haenszel test, df, degree of freedom.

Two studies[20, 21] reported the number of LNM cases between high and low expression of HULC for 404 cancer patients. Because of severe heterogeneity with 2 studies (*I*^*2*^ = 80%, *P*_*Q*_ = 0.03), the random-effects model was used to calculate the pooled effect. The LNM rate was significantly elevated in patients with high versus HULC expression, with pooled OR = 4.83 (95% CI 1.59–14.63) (Fig 2a).

#### Association between HULC and DM

Three studies[20, 19, 18] reported the number of DM cases for the 2 groups of HULC expression, for 216 patients. With no severe heterogeneity among the studies (*I*^*2*^ = 0%, *P*_*Q*_ = 0.93), the fixed-effects model was used in the pooled analysis. Risk of developing DM was increased with high versus low HULC expression (OR = 5.44, 95% CI 2.33–12.74) (Fig 2b).

### Association between HULC and prognosis

#### Association between HULC and OS

**Fig 3:**
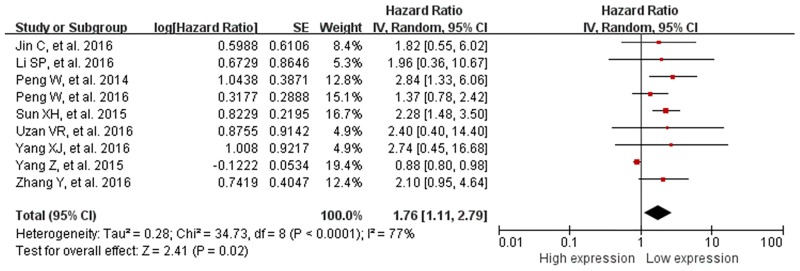
Forest plot of association between HUCL expression and overall survival (OS) under the random-effects model. Data are hazard ratios (HRs) and 95% CIs. **Abbreviations:** SE, standard error; IV, inverse variance methods; CI, confidence interval, df, degree of freedom.

All included studies showed data for OS by HULC level for 966 cancer patients. Because of significant heterogeneity (*I*^*2*^ = 77%, *P*_*Q*_<0.0001), the random-effects model was used. The pooled HR of OS was 1.76 (95% CI 1.11–2.79, *P* = 0.02) for high versus low HULC expression (Fig 3), so high HULC expression decreased the OS time. Because the detected specimens were from cancer tissues and serum of patients, we also calculated the pooled HR for OS by specimen type. For studies of cancer tissue [19, 11, 21, 13, 18, 23, 17], the pooled HR for OS was 1.72 (95% CI 1.01–2.92) under the random-effects model (*I*^*2*^ = 80%, *P*_*Q*_<0.0001) (S1a Fig) and for studies of serum, was 2.01 (95% CI 1.04–4.64) under the fixed-effects model (*I*^*2*^ = 0%, *P*_*Q*_ = 0.85) [20, 22] (S1b Fig).

#### Association between HULC and recurrence

**Fig 4:**
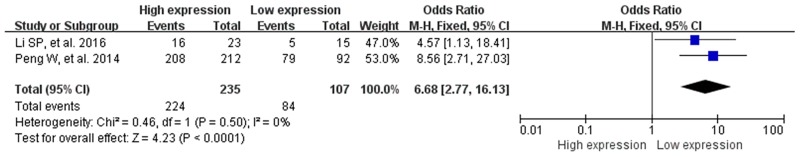
Forest plot of association between HULC expression and recurrence.

Two studies [19, 21] reported the number of recurrences by expression of HULC for 342 patients. The 2 studies showed no significant heterogeneity (*I*^*2*^ = 0%, *P*_*Q*_ = 0.50), and a fixed-effects model was used. High HULC expression significantly increased the recurrence of cancer in patients (OR = 6.68, 95% CI 2.77–16.13) (Fig 4).

### Sensitivity analysis

Sensitivity analysis was conducted for the association between HULC and OS. Each study was deleted in turn to examine the influence of the removed data on the overall HR (Table 3). The exclusion of each study did not change the significant result, which suggested that the pooled result was robust and not from random effects. Furthermore, when the results of Yang Z, et al. were removed from the pooled analysis, the degree of significance increased and no severe heterogeneity was found among the other studies (*I*^*2*^ = 0%, *P*_*Q*_ = 0.88). It implied that the heterogeneity among studies resulted from the Yang Z, et al. study and the effect of HULC on OS differed among ethnic groups. Therefore, we pooled the effect of HULC on OS for Chinese studies and found that high HULC expression significantly decreased the OS time under a fixed-effects model (HR = 2.04, 95% CI 1.55–2.68) (S2 Fig).

**Table 3:** Sensitivity analysis for the studies that resulted in loss of significance after omission.

Original summary effects				Omitted study	Resulting summary effects			
HR	95%CI	Nature of association	I^2^		HR	95%CI	Nature of association	I^2^
1.76	1.11–2.79	significant increased risk	77%	Jin C, et al.	1.76	1.08–2.87	significant increased risk	79%
				Li SP, et al.	1.75	1.09–2.83	significant increased risk	79%
				Peng W, et al. 2014	1.63	1.02–2.59	significant increased risk	74%
				Peng W, et al. 2016	1.87	1.09–3.21	significant increased risk	79%
				Sun XH, et al.	1.63	1.03–2.58	significant increased risk	64%
				Uzan VR, et al.	1.74	1.08–2.79	significant increased risk	79%
				Yang XY, et al.	1.72	1.07–2.77	significant increased risk	79%
				Yang Z, et al.	2.04	1.56–2.68	significant increased risk	0%
				Zhang Y, et al.	1.72	1.05–2.82	significant increased risk	78%

### Publication bias

**Fig 5:**
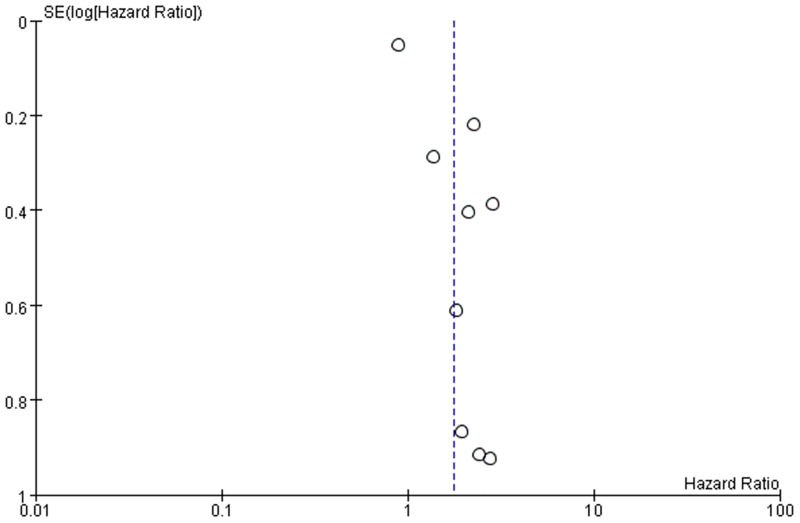
Begg’s funnel plot with pseudo 95% CIs testing publication bias with the association between HULC and OS.

Publication bias for the association between HULC and OS was checked by a Begg’s funnel plot under the random-effects model (Fig 5). Although the funnel plot seemed asymmetric, Begg’s test showed no significant rank correlation with Kendall score (*Z* = 0.31, *Pr*> |z| = 0.754). Given this result, we performed Egger’s test where evidence of significance publication bias was found (*r* = 1.90, 95% CI 1.01–2.79, *P>|t|* = 0.001). Therefore, studies finding high HULC expression associated with short OS might more frequently be published and included in the meta-analysis (S3 Fig). We performed trim and fill analysis to adjust the final effect; the HR 1.48 (95% CI 1.03–2.12) and *P* value for heterogeneity 0.004, showing a significant difference in the effect on OS among ethnic groups.

## Discussion

This current study aimed to assess the pooled effect of HULC expression on prognosis with cancer. High HULC expression was associated with increased cancer metastasis and decreased survival time. Furthermore, high HULC expression was an important factor in cancer recurrence. Because most of the studies were from China, the level of HULC can be useful for predicting prognosis for Chinese cancer patients and providing a target for therapy.

HULC, originally found in hepatocellular carcinoma, has been found highly expressed in tumor tissues in different cancer as compared with paired corresponding non-cancerous tissues. Moreover, silencing HULC could significantly inhibit tumor cell proliferation, migration and invasive capability in vitro. Overexpressing HULC could enhance cancer cell proliferation, migration and invasion.[24, 25, 18] Preliminary research in the mechanism of HULC found that it could co-purify with ribosomes in the cytoplasm and play a role in post-transcriptional modulation of gene expression.[26, 27] Research has identified that HULC could perturb the circadian rhythm of cancer cells and contribute to the epithelial-to-mesenchymal transition (EMT), required for cancer metastasis and invasion.[19, 27, 25, 24] In addition, HULC knockdown could enhance cisplatin-induced apoptosis in cancer cells. Therefore, HULC is an oncogenic lncRNA and participates in tumor development and progression.

In our meta-analysis, 4 studies reported the association between HULC expression and metastasis and 9 the effect of HULC on survival. The risk of occurrence of LNM and DM was increased with high versus low HULC expression and the OS time was shorter. Although subgroups of survival, such as EFS, DFS and PFS, could not be pooled because of too few studies, high HULC expression was still significantly associated with cancer recurrence. In addition, we examined the results for HULC tested in serum and tissue [20, 22, 28] and found OS time decreased with high versus low serum level of HULC. Because the effect of serum HULC level on OS was similar to that of tissue content, serum HULC level might be used for surveying the effect of therapy for cancer patients.

Some meta-analyses focused on the association of lncRNAs such as MALAT-1[29], AFAP1-AS1[30], H19[31], and PVT1[32] and metastasis as well as prognosis with cancer; all analyzed only the lncRNAs detected in cancer tissues. To search for an applicable biomarker for therapy, we focused on the association of tissue HULC content on metastasis and prognosis as well as serum HULC content and prognosis. To our best knowledge, this is the first meta-analysis of the effect of HULC on outcomes for cancer patients.

Our study contains some limitations. First, the types of cancer and samples of patients in the included studies were few and publication bias existed. Second, because of heterogeneity of ethnic groups in the included studies and most studies from China, the results may represent Chinese cancer patients only. Third, because of the nature of the meta-analysis using aggregated group data, the confounding factors could not be controlled. Fourth, because there were few studies on expression of HULC with LNM, DM recurrence, and OS in subgroup, significance of some of our findings was limited by the low precision as indicated by the wide confidence intervals. Therefore, larger-scale, multicenter, and high-quality studies are needed to confirm our findings.

## Conclusions

This meta-analysis is the first to demonstrate that high expression of the long noncoding RNA HULC is related to poor prognosis for cancer patients. The expression of HULC, especially tested in serum, might be a biomarker for surveillance of prognosis for cancer patients, especially in China.

## Supporting information

S1 Fig **a)**. Forest plot of pooled hazard ratios (HRs) for overall survival (OS) with highly upregulated in liver cancer (HULC) detected in tissue under the random-effects model. **b).** Forest plot of the pooled HRs for OS with HULC detected in serum under the fixed-effects model. **Abbreviations:** SE, standard error; IV, inverse variance methods; CI, confidence interval; df, degree of freedom.
(PNG)

S2 Fig Forest plot of the pooled HRs for OS for in Chinese under the fixed-effects model. (PNG)

S3 Fig Funnel plot with pseudo 95% confidence limits. (PNG)

S1 Table PRISMA 2009 checklist (HULC). (DOC)

## Abbreviations

DFS: disease-free survival
DM: distant metastasis
EFS: event-free survival
HR: hazard ratio
HULC: highly upregulated in liver cancer
LNM: lymph node metastasis
OR: odd ratio
OS: overall survival
PFS: progression-free survival
