# Supplementary figures and images for: AlncRNA HULC as an effective biomarker for surveillance of the outcome of cancer: A meta-analysis
Source: PLoS One. 2017 Feb 1;12(2):e0171210. doi: 10.1371/journal.pone.0171210 (PMC5287472; doi:10.1371/journal.pone.0171210)

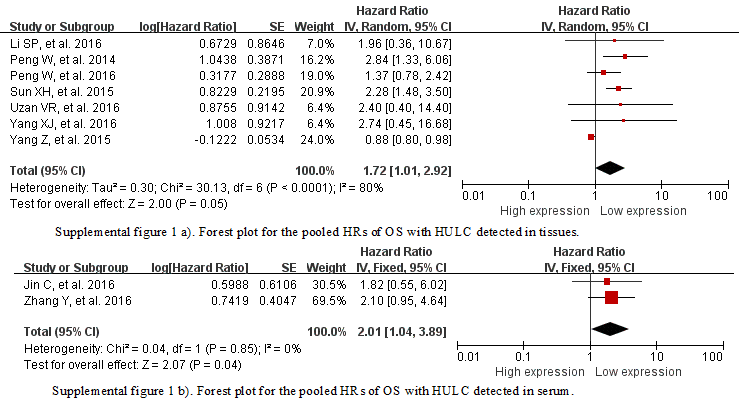

Supplement: S1 Fig — a). Forest plot of pooled hazard ratios (HRs) for overall survival (OS) with highly upregulated in liver cancer (HULC) detected in tissue under the random-effects model. b). Forest plot of the pooled HRs for OS with HULC detected in serum under the fixed-effects model. Abbreviations: SE, standard error; IV, inverse variance methods; CI, confidence interval; df, degree of freedom. (PNG) [file pone.0171210.s001.png]

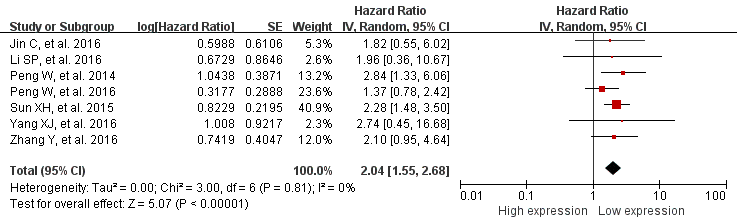

Supplement: S2 Fig — (PNG) [file pone.0171210.s002.png]

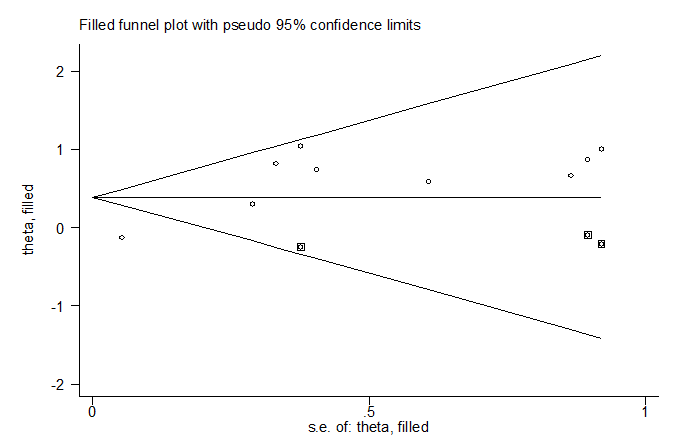

Supplement: S3 Fig — (PNG) [file pone.0171210.s003.png]
